# Evaluating the Psychometric Properties of the Arabic Version of the Appraisal of Self-Care Agency Scale-Revised (ASAS-R) Among Arabic Patients With Type 1 Diabetes Mellitus

**DOI:** 10.1155/nrp/8253318

**Published:** 2025-10-14

**Authors:** Muhammad W. Darawad, Basema Nofal, Ali M. Saleh, Elham Othman, Aziza Salem, Arwa Masadeh

**Affiliations:** ^1^School of Nursing, The University of Jordan, Amman 11942, Jordan; ^2^Faculty of Nursing, Al-Zaytoonah University of Jordan, Amman 11733, Jordan; ^3^School of Nursing, Applied Science Private University, Amman 11931, Jordan; ^4^Nursing Department, University of Tabuk, Tabuk, Saudi Arabia; ^5^School of Nursing, Al-Balqa Applied University, As-Salt, Jordan

**Keywords:** appraisal of self-care agency scale, Arabic, diabetes self-care agency, Jordan, psychometric properties

## Abstract

**Background:**

Self-care agency is crucial for the management of diabetes patients. The Appraisal of Self-Care Agency Scale-Revised (ASAS-R) is a reliable and brief measure of diabetic patients' SCA. In the MENA region, there is currently no Arabic version of the ASAS-R scale available.

**Aim:**

To evaluate the psychometric properties of the Arabic version of the ASAS-R among Arabic patients with DM.

**Methods:**

This study utilized a descriptive cross-sectional design. Exploratory factor analysis was used to test the scale's construct validity. Pearson correlation was used to test for its criterion-related and convergent validity.

**Results:**

Participants' ASAS-R total scores significantly correlated with diabetes self-efficacy (*r* = 0.543, *p* ≤ 0.001) and diabetes self-care management (*r* = 0.566, *p* ≤ 0.001) but did not correlate with their demographics. Factor analysis revealed a 2-factor solution that retained all items and explained a variance of 46.3%. Cronbach's alpha was 0.775 for the total scale and 0.691–0.851 for subscales indicating a high internal consistency. Also, no item redundancy was noted with the maximum interitem correlation of 0.695.

**Conclusion:**

The ASAS-R was found to be a psychometrically sound measure to evaluate self-care agency among Arabic patients with Type 1 diabetes mellitus, for which future studies interested in self-care among patients with diabetes are invited to use the ASAS-R to validate its psychometric properties.

## 1. Introduction

Diabetes mellitus (DM) is a debilitating, long-term condition affecting millions worldwide, with Type 1 diabetes mellitus (T1DM) posing a particular challenge for younger populations. According to the International Diabetes Federation (IDF), approximately 8.75 million people are currently living with T1DM globally, including 1.52 million individuals under the age of 20, and these numbers are projected to rise [1]. In 2021 alone, 530,000 new cases of DM were reported, with 201,000 among youth under 20. The World Health Organization [2] ranks DM as the ninth leading cause of death globally, reflecting its growing burden on public health.

The Middle East and North Africa (MENA) region bears a disproportionately high prevalence of DM, second only to other global regions, due to complex socioeconomic and demographic factors [3]. A recent IDF report highlighted that 1.9 million people with T1DM live in low- and lower-middle-income countries, including many in the MENA region [1]. In Jordan, a developing country in this region, DM prevalence is particularly high. Type 2 diabetes, for example, had a prevalence rate of 14.0% in 1990, increased to an estimated 16.0% in 2020, and is projected to reach 20.6% by 2050, consuming roughly 25.2% of the nation's total health expenditure [4]. Jordan also reports a total of 6344 individuals diagnosed with T1DM [1].

Diabetes is known with its chronic and progressive nature, where patients were found to have a high percentage of anxiety and depressive symptoms, poor self-efficacy, moderate social support, and low self-care management [5]. Affected individuals must engage in continuous self-care to minimize complications and improve health outcomes. Effective self-care involves complex daily tasks such as blood glucose monitoring, insulin administration, meal planning, and physical activity [6, 7]. However, numerous physical and psychological barriers may hinder patients' ability to sustain these behaviors [8, 9]. Therefore, understanding and assessing an individual's capacity to engage in self-care is essential.

Orem's Self-Care Deficit Nursing Theory [10] provides a theoretical foundation for conceptualizing self-care behaviors. According to Orem, self-care refers to actions individuals perform on their own behalf to maintain health and well-being. The ability to perform these actions—termed self-care agency (SCA)—is a learned capacity that develops over time. Orem defined SCA as “the acquired, complex capacity to meet the requirements to take care of oneself, regulating life processes, and maintaining or promoting integrity, structure, and functioning, as well as one's development and promotion of well-being” (p. 20). SCA comprises three categories of personal characteristics: foundational, enabling (power components), and operational. In the context of chronic conditions like DM, SCA is a critical determinant of a patient's ability to effectively manage their illness.

Evidence supports the relationship between SCA and improved health outcomes. Research has shown that higher levels of SCA are associated with better glucose control, medication adherence, and quality of life [11, 12]. Among patients with T1DM, Rahmani et al. [13] found a significant positive correlation between SCA and quality of life. Similar findings were reported among the general population, with Damasio and Koller [14] and Oliveira et al. [15] demonstrating that SCA is linked to subjective happiness, life satisfaction, and mental well-being.

Given the importance of SCA, accurate and culturally sensitive tools are needed to assess this construct in clinical and research settings. Several self-report instruments have been developed to measure SCA, including Lorensen's Self-Care Capability Scale [16], Denyes' Self-Care Agency Scale [17], and the Perception of Self-Care Agency Questionnaire [18]. Among these, the Appraisal of Self-Care Agency Scale-Revised (ASAS-R) has emerged as a brief and psychometrically sound measure grounded in Orem's theory. The ASAS-R consists of 15 items covering three dimensions: having power for self-care, developing power for self-care, and lacking power for self-care. The scale has demonstrated good internal consistency (*α* > 0.83) and has been validated across diverse populations [7].

The ASAS-R has been translated and validated in multiple languages. For example, the Portuguese version demonstrated a 3-factor structure explaining 53.9% of the variance and yielded Cronbach's alpha values ranging from 0.55 to 0.85 [15]. Similarly, the Farsi version showed good reliability (*α* = 0.76) and significant correlations with HbA1c levels, quality of life, and illness duration among patients with T1DM (Rahmani et al., 2018). Other successful adaptations have been reported in Brazilian [14] and Chinese [19] contexts, further supporting the scale's cross-cultural applicability.

Despite the growing interest in self-care assessment and the widespread burden of DM in the MENA region, no validated Arabic version of the ASAS-R currently exists. This gap is particularly pressing given the need for culturally adapted psychosocial instruments for Arabic-speaking populations living with chronic illnesses, including diabetes [20, 21]. Moreover, Sousa et al. [7] emphasized the need for further psychometric evaluation of the ASAS-R in diverse populations.

In response to this critical need, the present study aims to translate and validate the Arabic version of the Appraisal of Self-Care Agency Scale-Revised (ASAS-R-AR). Establishing a psychometrically sound Arabic instrument will support individualized assessment of self-care ability in clinical practice and enable further research among Arabic-speaking patients with diabetes.

## 2. Methods

### 2.1. Design and Setting

As a component of a broader research initiative, this investigation employed a descriptive cross-sectional approach by conducting an online survey using Google Forms. The use of an online survey was primarily driven by participant convenience and resource limitations. The respondents were recruited from two large healthcare institutions (a public and a teaching hospital) situated in Amman and Irbid, the most populous cities in Jordan.

### 2.2. Sampling

The scope of this study focuses on Jordanian individuals diagnosed with TDM, with the accessible population comprising patients attending the diabetes outpatient clinics at the chosen hospital. The study sample was obtained through a convenience sampling technique. To be eligible for participation, participants had to meet the following criteria: (1) aged between 18 and 30 years, (2) diagnosed with T1DM for at least six months, and (3) capable of completing the online survey via a smartphone. However, individuals with severe physical, mental, or cognitive impairment were excluded from the study. Sample size was determined using G-power 3.1.9.2, and using power of 0.8, medium effect size of 0.25, and level of significance of 0.05 and ANOVA, the minimum total sample size would be 128.

### 2.3. Instruments

In the present study, a set of assessment tools was employed, beginning with the collection of demographic information from the selected participants, including age, gender, education level, marital status, employment status, income, smoking habits, comorbidities, and whether they received health education about DM. To achieve the study's objectives, three scales were utilized, which were translated from their original English version to Arabic following the WHO [22] translation guidelines. The scales were initially forward-translated into Arabic, followed by back-translation into English by an independent translator. Lastly, pilot-testing was conducted to establish the final version of the scales.

The ASAS-R [7] was the primary scale used to assess participants' SCA in managing their disease. It includes 15 items across three subscales—having, developing, and lacking power—rated on a 5-point Likert scale (1 = *totally disagree* to 5 = *totally agree*). After reverse scoring negatively worded items, total scores range from 15 to 75, with higher scores indicating greater SCA. Construct validity was supported by factor analysis revealing a 3-factor model explaining 61.7% of item variance. Internal consistency was strong, with Cronbach's alpha of 0.89 for the total scale and 0.79–0.86 for subscales. This revised version followed the original ASAS [23], which showed a single factor among American adults with diabetes and adequate psychometrics. The 3-factor structure of ASAS-R was also confirmed in the Portuguese version tested on medical students [15], with Cronbach's alpha ranging from 0.55 to 0.85 across subscales. The scale has been validated in both Southeast Asian and European contexts, with Santos [24] confirming its validity in the Philippines and Topi et al. [25] demonstrating strong reliability in a Greek population.

The second instrument, the Diabetes Self-Efficacy Scale (DSES) [23], assesses individuals' confidence in performing diabetes management behaviors. It comprises 60 items across seven domains: healthy eating, physical activity, blood glucose monitoring, insulin adherence, foot care, problem solving, and risk reduction. Responses are recorded on a 6-point Likert scale (0 = *strongly disagree* to 5 = *strongly agree*), yielding a total score range of 0 to 300, with higher scores reflecting greater self-efficacy. Validity was confirmed through content analysis, with a content validity index of 0.97 for the entire scale and item-level indices ranging from 0.8 to 1.0.

The final instrument used was the Diabetes Self-Management Scale (DSMS), which assesses patients' actual performance of diabetes self-care behaviors based on Orem's Theory of Self-Care [23]. The DSMS includes the same 60 items and subscales as the DSES, using the same scoring system (0–300), where higher scores indicate greater adherence to self-care activities and better self-management. Content validity was confirmed by the original authors, with a scale-level index of 0.96 and item-level indices above 0.78. The DSMS has been recently validated across different populations, with Dai et al. [26] confirming its reliability in Chinese patients with Type 2 diabetes and Kaabi et al. [27] demonstrating strong psychometric properties for the Arabic version among Arabic-speaking individuals.

### 2.4. Data Collection

Once ethical approvals were obtained, a member of the research team (AM) visited the diabetes clinic at the participating hospitals to meet with the nurse manager and explained the purpose of the study. She sought permission to meet with potential participants, who were interviewed to determine their eligibility to take part in the study. Eligible participants were then asked to sign an informed consent form, which explained the purpose of the study and their rights as participants. The study's online survey link was then sent to their provided phone numbers, which contained the study package to be completed during their waiting time at the clinic. The survey took approximately 15–20 min to complete, during which the data collector was available to respond to any concerns or questions the participants had. To ensure data quality and prevent duplicate responses, we (1) limited the survey to one response per device; (2) included a required consent question to confirm participation; and (3) reviewed responses for completeness and consistency before analysis.

A pilot study was conducted among 15 participants to assess the feasibility of the study, the comprehensibility of the study instruments, technical issues related to the online link, and to estimate the time required to complete the online survey. Feedback from participants indicated that the items were generally clear and easy to understand, with minimal issues related to wording or interpretation leading to minimal changes accordingly. Also, Test–retest reliability was assessed with a subsample of 20 participants, using a 2-week interval between the initial and follow-up administrations to evaluate the temporal stability of the instrument.

### 2.5. Ethical Considerations

This study protocol was ethically reviewed and approved by the Scientific Research Committee at School of Nursing-the University of Jordan and the Ministry of Health. Measures taken to protect participant confidentiality and data privacy included obtaining informed consent, anonymizing responses, and anonymous data collection through avoidance of collecting personally identifiable information, and ensuring secure data storage. For the online survey, features in used platform were configured to prevent IP tracking and duplicate submissions, and data access was restricted to the research team only.

### 2.6. Data Analysis

The Social Package for Social Sciences (SPSS), version 25, was used to run data analysis considering a level of significance < 0.05. Evaluating the psychometric properties of the ASAS-R-AR in this study was possible through using adequate sample size, which was tested using the Kaiser–Meyer–Oklin measure. The normality of the total ASAS-R-AR score was first assessed using the Kolmogorov–Smirnov test, which indicated approximate normality and thereby supported the use of maximum likelihood extraction. Also, descriptive statistics (means and standard deviations) were used to describe the items and subscales of the ASAS-R-AR (15 items) and the rest of the study variables.

Starting with the construct validity, it was tested using Gorsuch [28] guidelines for data reduction by running exploratory factor analysis (EFA) including the 15 items of the ASAS-R-AR scale, utilizing principal axis component and considering the oblique promax rotation, assuming that factors are correlated after confirming the intercorrelation between the items of the scale [29]. The EFA was used because this study is the first to apply this instrument among Arabic patients. Also, the following parameters were set as a cut-off point including ≥ 0.40 for a significant loading on a factor, ≥ 0.90 for interitem correlation to avoid possible item redundancy [30], and 10% of common variance to be achieved by a factor to be considered supported by a scree plot observing the natural breaks in the factors.

To assess the criterion-related validity of the ASAS-R-AR scale, the Pearson correlation test was used to examine the correlation between the total scores of the scale and subscales with participants' scores on the DSMS scale and selected demographic and clinical variables such as age, income, and duration of diabetes. Also, the Pearson correlation test was used to assess the convergent validity of the ASAS-R-AR by assessing the scale and subscales total scores' correlation with participants' total scores on DSES. Conceptually, DSMS reflects actual behaviors and DSES captures self-efficacy beliefs, together providing complementary behavioral and psychological perspectives that support the validity of the ASAS-R. Additionally, to determine the internal consistency of the ASAS-R-AR scale and its subscales, Cronbach's alpha was calculated. Finally, to assess the stability of the ASAS-R-AR, a test–retest reliability was estimated using the Pearson correlation test.

## 3. Results

A total of 128 participants accepted participation. As shown in Table 1, the majority of the sample were males (58.6%, *n* = 75), singles (74.2%, *n* = 95), educated with less than high school (46.9%, *n* = 60), unemployed (67.2%, *n* = 86), with an income less than 500 JOD (73.5%, *n* = 92), nonsmokers (69.5%, *n* = 89), had no DM comorbidities (58.6%, *n* = 75), and reported receiving health education about DM (91.4%, *n* = 117). Further, their mean age was 23.4 years (SD = 4.09), with a mean duration of disease of 115.5 months (SD = 80.1). Regarding the major study variables, participants reported a relatively high level of SCA that was 74.1% (*M* = 55.5/75, SD = 6.87), high level of self-care efficacy that was 71.8% (*M* = 215.46/300, SD = 34.99), and high level of self-care management that was 68.1% (*M* = 204.16/300, SD = 40.35).

### 3.1. Testing Reliability of the ASAS-R-AR

Test–retest reliability was calculated to assess scale stability, which was found to be 0.623 indicating a stable scale over time. To assess the internal consistency reliability of the ASAS-R-AR, Cronbach's alpha (*α*) was calculated for its total scale and subscales, considering the minimum cut-off point (0.70) determined by Polit and Beck [31]. Results revealed the alpha value of 0.775 for the total scale, while it was 0.851 for the having/developing power and 0.691 for the lacking power indicating a high internal consistency for this scale. Also, alpha if-item-deleted was calculated (Table 2), which ranged from 0.746 to 0.804 indicating no items could be deleted to increase the internal consistency. As seen in Table 3, the range for mean interitem correlation ranged from 0.11 to 0.695 (having/developing power) and 0.228 to 0.659 (lacking power). As none reached a correlation of 0.70, it can be concluded that there is no item redundancy. Also, it was noted that items 1 and 2 had the highest interitem correlation (*r* = 0.695) followed by the correlation between items 14 and 15 (*r* = 0.659).

### 3.2. Testing Validity of the ASAS-R-AR

To test for the construct validity of the ASAS-R-AR, the EFA was utilized using principal component analysis and oblique promax rotation. The Kaiser–Meyer–Oklin Measure of Sampling Adequacy was found to be 0.788, indicating adequate sampling consistent with Bartlett Test of Sphericity (approximate chi-square = 642.02, *p* ≤ 0.001). Contrary to the 3-factor model in the original scale, results of the factor analysis revealed a 2-factor solution to the data that was supported by the scree plot, with an eigenvalue of 8.3 and an explained variance of 46.3%. Further, all items of the ASAS-R-AR were retained in the factor analysis as all of them had a factor loading greater than 0.40 (Table 2), with no cross-loaded items except three items. Concerning items distribution, 11 items loaded on the having/developing power factor (items of having power and developing power in the original scale) and four items loaded on the lacking power factor (same items of lacking power in the original scale).

The item that had the highest loading (0.799) was item number 14 “*I seldom have time for myself*,” followed by item number 3 “*When needed, I set new priorities in the measures that I take to stay healthy*” with a loading of 0.775. On the other side, the item that had the lowest loading (0.517) was item number 7 “*If I take a new medication, I obtain information about the side effects to better care for myself*” preceded by item number 12 “*I am able to get the information I need, when my health is threatened*” with a loading of 0.528.

The criterion-related validity of the ASAS-R-AR was assessed by its correlation with DSMS. The results (Table 4) showed a significant positive correlation (*r* = 0.57, *p* ≤ 0.001). Also, the two ASAS-R-AR subscales were found to have significant positive correlation with DSMS scores including having/developing power (*r* = 0.58, *p* ≤ 0.001) and lacking power (*r* = 0.181, *p* < 0.01). However, the correlation magnitude was moderate with having/developing power and weak with lacking power. The variation in magnitude of the correlation indicates the methodological direction of ASAS-R-AR being associated with positive rather than negative factors. Regarding the convergent validity (Table 4), participants' DSES scores had significant positive correlation with their scores on ASAS-R-AR total (*r* = 0.54, *p* ≤ 0.001) and having/developing power (*r* = 0.58, *p* ≤ 0.001), with no significant correlation with the lacking power. However, it was noted that neither the ASAS-R-AR total scale nor its subscales scores had significant correlation with participants' demographics except a significant negative correlation between age and lacking power (*r* = 0.191, *p* < 0.01).

## 4. Discussion

The purpose of this study was to evaluate the psychometric properties of the Arabic version of the ASAS-R aiming to add proof to the usability of this scale to promote healthcare providers' clinical assessment of SCA among patients with T1DM, which would help in tailoring individualized care plan considering patients' self-care capabilities [7]. Also, such a study would enrich the body of knowledge concerning the psychosocial behaviors of patients with DM and meanwhile add an additional valid and reliable Arabic tool to be used by researchers in the Arab world who are interested in health promotion among Arabic patients having various chronic diseases. Despite the relatively small sample size used in this study, the measure of sampling adequacy indicated adequate sampling. Besides, participants were recruited from two large hospitals in two different regions of Jordan, which supports the representativeness of the sample and the generalizability of the study finding in terms of the utilization of the ASAS-R-AR in future Arabic studies in this field.

The results of testing the construct validity of the ASAS-R-AR revealed a 2-factor solution with an explained variance of 46.3%, which is contrary to the 3-factor model in the original scale [7] and the various translated versions in Brazil [14], Portugal [15], and China [19] that had a slightly better explained variance (54.5%–61.7%). One explanation of such discrepancy is knowing that the ASAS was a single-factor scale developed among patients with DM [23] that later was revised among a sample of the general population [7]. Our study recruited a sample of diabetic patients who had a relatively similar duration of illness used in the study of Sousa et al. [23] indicating that participants in both studies have already developed their own self-care capabilities leading to less factors to explain the variance. The rest of the abovementioned studies were conducted among medical students [15], older people [19], and general population [14], all of which may not have experienced chronic illnesses, and they are still developing their behaviors of health promotion [7]. Previous studies that examined the validity of ASAS reported a 7-factor scale among elderly people in Hong Kong [32] and a 5-factor scale among home-dwelling elderly in Sweden [33], which suggests the high effect of the cultural diversity that should be considered while applying this scale. To further strengthen our argument, we assessed the criterion-related validity of the ASAS-R-AR by examining its correlation with the DSMS, which was found to have significant positive correlations with the total scale, as well as its two subscales. While this supports the theoretical relevance of the identified factors within our sample, deeper exploration of their conceptual meaning would require additional qualitative or mixed-method research, which we suggest for future studies.

In the EFA, all items of the ASAS-R-AR were included as they had a factor loading greater than 0.40, except for three items that had cross-loaded. Interestingly, these three items belonged to the Developing Power subscale, which was also observed in the 3-factor solution of the original revised scale [7]. This finding could be explained by the young age of the participants (18–30 years), who might have had difficulty distinguishing between having power and developing power, as these three items referred to being in a “bad situation,” such as an inability to take care of oneself and threats to one's health. However, other studies reported different items loading on different subscales, indicating that cultural factors can influence how the scale items are perceived [14, 15]. Therefore, our results support the usability of the ASAS-R-AR, which opens the door for future Arabic studies in different Arabic countries to use this measure and evaluate its psychometric properties among various groups of participants. Further, it is suggested to conduct qualitative follow-up research to explore how cultural perceptions may shape the interpretation of self-care agency items, which could further enhance the scale's cultural relevance and applicability.

The ASAS-R-AR total scale and subscales demonstrated significant positive correlations with participants' scores on self-efficacy and diabetes self-management, providing evidence for the instrument's convergent and criterion-related validity. This finding is supported by previous studies that have shown significant correlations between ASAS-R total and subscale scores and various variables such as quality of life (Rahmani et al., 2018), subjective happiness, satisfaction with life, mental health [15], and exercise of SCA [19]. The moderate positive correlations observed between the ASAS-R-AR and both the diabetes self-efficacy and self-management scales (*r* ≈ 0.55) support the scale's convergent validity, while also indicating that self-care agency represents a related yet distinct construct. This aligns with theoretical expectations and reinforces the conceptual uniqueness of self-care agency in the context of diabetes management. Therefore, having a standardized, valid, and reliable scale such as the ASAS-R-AR can help healthcare providers better understand the self-care capabilities of diabetic patients, ultimately improving patient outcomes.

On the other hand, the ASAS-R-AR total scale and subscales did not show any significant correlation with participants' demographic characteristics, which is in contrast to previous studies that found significant correlations with factors such as duration of illness, glycated hemoglobin, age, income, and education level Rahmani et al., 2018; [14]. The reasons for this inconsistency with the literature are uncertain, but it could be due to the small sample size and the young age of the participants. It is essential to investigate indicators of patients' self-care capabilities in managing T1DM, a complex and demanding disease, to promote their health-related behaviors. Therefore, it is suggested that future studies should explore potential moderators or mediators that might explain demographic influences. Nurses play a crucial role in this regard; however, they have been found to have inadequate knowledge and attitudes towards DM [34], which may prevent them from providing appropriate support to their patients. Therefore, healthcare providers involved in DM care should be updated on how to deal with their patients and assess their needs, which was found to be helpful in this regard [35].

The ASAS-R-AR's reliability was evaluated using test–retest correlation to assess its consistency over time, and it was found to be 0.62 which indicates a stable scale. This finding was supported by a study done by Guo et al. [19] which reported a test–retest correlation of 0.95 among the Chinese population. The observed discrepancy in test–retest reliability between our study (*r* = 0.62) and the previously reported value in the Chinese sample (*r* = 0.95) is notable. This variation may be attributed to cultural differences in response patterns, the length of the test–retest interval, or other methodological factors such as sample size or administration mode. Further psychometric validation studies are recommended to confirm the temporal stability of the ASAS-R-AR across diverse contexts and populations. In terms of internal consistency, Cronbach's alpha for the total scale was 0.78, while it was 0.85 for the having/developing power subscale and 0.69 for the lacking power subscale, indicating high internal consistency for this scale. However, it is noteworthy that Cronbach's alpha for the “lacking power” subscale approaches the lower threshold of acceptability, which may be attributed to the limited number of items or variability in participants' interpretation, for which we suggest further refinement or cultural adaptation of the subscale would improve its internal consistency in future research.

Most previous studies reported similar values of Cronbach's alpha for the total scale, including 0.89 for the original English version [7], 0.85 for the Portuguese version [15], and 0.79 for the Chinese version [19]. Interestingly, the subscales of ASAS-R showed acceptable Cronbach's alpha values except for developing power, which was reported as unsatisfactory in two studies, the Portuguese version (0.55) among medical students [15], and the Brazilian version (0.38) among patients with DM [36]. This implies that the application of this factor among patients with chronic illnesses such as DM may pose some issues. However, our results suggest that the ASAS-R-AR is a reliable scale for evaluating the self-care capabilities of Arabic patients with DM, as indicated by the high internal consistency.

### 4.1. Strengths and Limitations

To our knowledge, this study is the first to evaluate the Arabic version of the ASAS-R that assessed SCA among Arabic patients with T1DM. The use of this valid and reliable scale encourages future research to identify determinant factors of patients' SCA, correlate it with other psychosocial and clinical variables, and evaluate the impact of using ASAS-R-AR as a screening tool on diabetic patients' outcomes. Also, it is recommended to evaluate the psychometric properties of ASAS-R-AR among various Arabic populations in different Arabic countries using a larger sample size.

Despite the numerous strengths of the current study, several limitations should be considered when interpreting its findings. Although the KMO test confirmed sampling adequacy, the relatively small sample size may have limited the statistical power of the factor analysis and the generalizability of results. The use of convenience sampling may also introduce selection bias, as participants with greater interest in or access to care may have been overrepresented. Additionally, the study focused exclusively on individuals aged 18–30 years, excluding younger adolescents and older adults with T1DM, whose self-care agency profiles may differ. The sample was drawn solely from Jordan, which, while valuable, limits generalizability to other Arabic-speaking populations; cultural differences may influence self-care perceptions and responses to the ASAS-R-AR across the Arab region. The cross-sectional design restricts conclusions about causality and temporal changes in self-care agency; therefore, longitudinal studies are needed to assess the scale's stability and responsiveness over time. Also, while administering the survey during clinic waiting times was practical and convenient, we acknowledge it may have caused some participants to feel rushed, potentially affecting data quality, for which future studies are suggested to allow more flexible completion settings.

Furthermore, the absence of clinical indicators such as HbA1c levels, insulin regimen, and diabetes complications limited the ability to assess criterion validity. Future research should integrate biomedical markers to contextualize self-care agency in relation to clinical outcomes. The reliance on a self-administered online survey may have introduced response bias, including social desirability bias, despite efforts to ensure anonymity. Lastly, while the ASAS-R-AR showed significant correlations with self-efficacy and self-management, other constructs—such as depression, health literacy, and social support—were not assessed. Future studies should explore these domains and investigate cross-cultural variations in factor structure to strengthen the instrument's validity.

## 5. Conclusion

This study aimed to assess the psychometric properties of the Arabic version of the ASAS-R in Jordanian patients with T1DM. The goal was to have a reliable and valid tool for evaluating patients' self-care capabilities. The results showed that the ASAS-R-AR was valid and reliable, as it correlated well with self-efficacy and self-management, and had acceptable values of Cronbach's alpha and test–retest correlation. The validated ASAS-R-AR scale holds significant potential for clinical application, as it can aid healthcare providers in identifying patients' SCA levels, facilitating more effective patient–provider communication, and guiding the development of culturally sensitive, individualized self-care interventions to improve diabetes management outcomes in Arabic-speaking populations. Also, despite some cultural variations and limitations, the study suggests that the ASAS-R-AR is a useful tool for assessing self-care capabilities among Arabic patients with T1DM, which can be utilized for future studies and programs to improve clinical outcomes.

## Figures and Tables

**Table 1:** Description of sample demographic variables (*N* = 128).

Variable	Range	Mean (SD)	(*n*) %
Gender			
Male			53 (41.4)
Female			75 (58.6)
Marital status			
Single			95 (74.2)
Married			33 (25.8)
Educational level			
Secondary or less			60 (46.9)
Diploma			13 (10.2)
Bachelor or higher			55 (42.9)
Level of employment			
Unemployed			86 (67.2)
Full time employment			28 (21.9)
Part time employment			14 (10.9)
Income			
Less than 300 JD			48 (38.3)
300–500 JD			44 (35.2)
501–1000			20 (15.6)
More than 1000			14 (10.9)
Smoking			
Yes			39 (30.5)
No			89 (69.5)
Comorbidities associated with diabetes			
No			75 (58.6)
Yes			53 (41.4)
Receiving health education about diabetes			
Yes			117 (91.4)
No			11 (8.6)
Age (years)	18–30	23.41 (4.09)	
Duration of disease (months)	6–312	115.5 (80.1)	
Self-care agency	30–74	55.54 (6.87)	
Self-care efficacy	97–299	215.46 (34.99)	
Self-care management	97–299	204.16 (40.35)	

**Table 2:** Factor analysis of ASAS-revised scale with item-loading on each factor.

Item #	Statement	ASAS-R dimensions (*α*)	Factor 1	Factor 2	*α* if item deleted
1	As circumstances change, I make the needed adjustments to stay healthy	Having/developing power (0.851)	0.742		0.747
2	If my mobility is decreased, I make the needed adjustments		0.739		0.750
3	When needed, I set new priorities in the measures that I take to stay healthy		0.775		0.746
5	I look for better ways to care for myself		0.539		0.765
6	When needed, I manage to take time to care for myself		0.640		0.755
7	If I take a new medication, I obtain information about the side effects to better care for myself		0.517		0.767
8	In the past, I have changed some of my old habits in order to improve my health		0.653		0.748
9	I routinely take measures to insure the safety of myself and my family		0.573		0.759
10	I regularly evaluate the effectiveness of things that I do to stay healthy		0.704		0.753
12	I am able to get the information I need, when my health is threatened		0.528		0.761
13	I seek help when unable to take care of myself		0.622		0.761

4	I often lack the energy to care for myself in the way that I know I should	Lacking power (0.691)		0.626	0.793
11	In my daily activities, I seldom take time to care for myself			0.621	0.804
14	I seldom have time for myself			0.799	0.766
15	I am not always able to care for myself in a way I would like			0.753	0.756

**Table 3:** Interitem correlation matrix.

	ASAS-1	ASAS-2	ASAS-3	ASAS-4	ASAS-5	ASAS-6	ASAS-7	ASAS-8	ASAS-9	ASAS-10	ASAS-11	ASAS-12	ASAS-13	ASAS-14	ASAS-15
ASAS-1	1.000														
ASAS-2	0.695	1.000													
ASAS-3	0.588	0.547	1.000												
ASAS-4	0.004	0.032	−0.073	1.000											
ASAS-5	0.336	0.296	0.371	−0.084	1.000										
ASAS-6	0.377	0.463	0.438	−0.177	0.343	1.000									
ASAS-7	0.244	0.232	0.353	−0.216	0.269	0.266	1.000								
ASAS-8	0.466	0.447	0.466	0.090	0.183	0.335	0.283	1.000							
ASAS-9	0.396	0.471	0.371	0.052	0.235	0.272	0.189	0.428	1.000						
ASAS-10	0.404	0.397	0.493	−0.004	0.315	0.379	0.370	0.343	0.414	1.000					
ASAS-11	−0.099	−0.166	−0.183	**0.228**	−0.141	−0.088	−0.126	−0.034	−0.083	−0.208	1.000				
ASAS-12	0.254	0.318	0.220	−0.075	0.283	0.276	0.283	0.371	0.110	0.396	−0.018	1.000			
ASAS-13	0.326	0.227	0.453	−0.201	0.385	0.349	0.363	0.281	0.214	0.491	−0.181	0.443	1.000		
ASAS-14	0.171	0.105	0.140	**0.263**	0.003	0.122	0.047	0.128	0.074	−0.027	**0.413**	0.101	0.018	1.000	
ASAS-15	0.211	0.204	0.259	**0.337**	0.045	0.201	0.080	0.239	0.116	0.174	**0.232**	0.082	0.058	**0.659**	1.000

*Note:* Bold indicates correlations of items of lacking power factor.

**Table 4:** Correlation of ASAS-revised scores with participants' demographics and other study variables.

	Age	BMI	Illness duration	Self-care efficacy	Self-care management
ASAS-R factor 1: Having/developing power	0.101	−0.105	−0.093	0.577^∗∗^	0.557^∗∗^
ASAS-R factor 2: Lacking power	−0.191^∗^	−0.099	−0.159	0.097	0.181^∗^
ASAS-R: Total score	−0.102	−0.137	−0.085	0.543^∗∗^	0.566^∗∗^

^∗^Significant at *p* < 0.05.

^∗∗^Significant at *p* < 0.01.

## Data Availability

The data that support the findings of this study are available on request from the corresponding author. The data are not publicly available due to privacy or ethical restrictions.
